# Evidence-Based Guideline on the Prevention and Management of Perioperative Pain for Breast Cancer Peoples in a Low-Resource Setting: A Systematic Review Article

**DOI:** 10.1155/2023/5668399

**Published:** 2023-11-03

**Authors:** Tajera Tageza Ilala, Gudeta Teku Ayano, Yesuf Ahmed Kedir, Selam Tamiru Mamo

**Affiliations:** Department of Anesthesia, College of Medicine and Health Science, Hawassa University, Hawassa, Ethiopia

## Abstract

**Background:**

Breast surgery for breast cancer is associated with significant acute and persistent postoperative pain. Surgery is the primary type of treatment, but up to 60% of breast cancer patients experience persistent pain after surgery, and 40% of them develop acute postmastectomy pain syndrome. Preoperative stress, involvement of lymph nodes while dissecting, and the postoperative psychological state of the patients play vital roles in managing the postoperative pain of the patients. The objective of this study is to develop evidence-based guideline on the prevention and management of perioperative pain for breast cancer surgical patients.

**Methods:**

An exhaustive literature search was made from PubMed, Cochrane Review, PubMed, Google Scholar, Hinari, and CINAHIL databases that are published from 2012 to 2022 by setting the inclusion and exclusion criteria. After data extraction, filtering was made based on the methodological quality, population data, interventions, and outcome of interest. Finally, one guideline, two meta-analyses, ten systematic reviews, 25 randomized clinical trials and ten observational studies are included in this review, and a conclusion was made based on their level of evidence and grade of recommendation.

**Results:**

A total of 38 studies were considered in this evaluation. The development of this guideline was based on different studies performed on the diagnosis, risk stratification and risk reduction, prevention of postoperative pain, and treatments of postoperative pain.

**Conclusion:**

The management of postoperative pain can be categorized as risk assessment, minimizing risk, early diagnosis, and treatment. Early diagnosis is the mainstay to identify and initiate treatment. The perioperative use of a nonpharmacological approach (including preoperative positive inspirational words and positive expectation) as an adjunct to the intraoperative regional anesthetic technique with general anesthesia with proper dosage of the standard pharmacological multimodal regimens is the first-line treatment. For postoperative analgesia, an extended form of intraoperative regional technique, nonpharmacologic technique, and NSAIDs can be used with the opioid-sparing anesthesia technique.

## 1. Introduction

Cancer is a condition in which aberrant cells (transformed tumor cells) can infect neighboring tissues and divide out of control [1]. Nearly 10 million people died from cancer worldwide in 2020, making it the top cause of death [2].

Breast cancer is the second-most common cause of cancer deaths next to cervical cancer. Women with BC have an 82% 5-year survival rate in Europe, compared to 46% in Uganda, 39% in Algeria, and 12% in Gambia [3, 4]. It is the most common malignancy among women worldwide, and in the majority of African nations, it account for one in four newly diagnosed cancer cases and one in five cancer-related fatalities among women in Africa [5, 6].

The incidence of BC varies significantly around the globe with higher incidence in the sub-Saharan countries. A variety of factors such as better healthcare, late marriage, first pregnancies, a decline in breastfeeding, sedentary lifestyle like increased tobacco use and decreased physical activity, and unhealthy diets consisting of fatty fast food may contribute to the continues increments of breast cancer [5, 7].

It may be managed by early diagnosis and the timely initiation of proper treatment including surgery, chemotherapy, radiotherapy, different hormonal therapies, or a combination of treatment modalities. The majority of breast cancer patient present at advanced stages (III and IV) at the time of their initial medical consultation, which makes surgery the main option of management with or without chemotherapy and radiation therapy [8]. This might be due to the reduced awareness, ignorance, and social variables like shyness, fear of the stigma associated with divorce, and a primary physician's low index of suspicion [7].

Breast surgery induces a considerable postoperative pain, whether an acute or chronic pain. It is reported that up to 60% of women experience chronic pain, and 40% experience acute postoperative pain following breast cancer surgery [9, 10].

Hence, inadequate postoperative pain management increases the perils of bad clinical outcomes, such as cardiac impairments, delayed mobilization, extended hospital stays, sleep disruptions, psychological stress, an increase in inflammatory cytokines, and sympathetic nervous system activation. In addition, it may be changed to chronic pain lasting for many years, and greatly affect the quality of life secondary to depression from breast resection, which may affect the physical, psychological, and socio-economic welfare of the patient [1, 11].

Recently, the preventive and management efforts are concentrated on the prevention of postoperative pain and making an early diagnosis and management. Nonpharmacological techniques, pharmaceutical analgesics, oral and intravenously, as well as more invasive procedures using local anesthetics, such as local anesthetic infiltration, intercostal block, thoracic epidural analgesia, and paravertebral block, are useful to control pain [4, 12].

The effective management of postoperative pain depends on the application of proper preoperative preventive complementary medicine and the institution of proper pharmacological techniques. Since the use of prescribed pharmaceuticals might not be adequate to address the pain effectively and decrease the patient's anxiety and distress, the use of nonpharmacological interventions as a multimodal component is strongly advocated. Hence, this needs multidisciplinary team collaboration and the development of comprehensive and integrated practical guidelines to insight the uniform clinical protocol throughout patient management. The current study generate updated evidence and clinical recommendation on the use of nonpharmacological (complementary medicine) including preoperative positive inspirational words and positive expectations, acupuncture, massage, and sleep in combination with the pharmacological approaches for the prevention and management of perioperative pain in breast cancer surgical patients for a low-resource area.

Therefore, the objective of this study is to develop an evidence-based practice guideline on the prevention and management of perioperative pain for breast cancer surgical patients in a low-resource area.

## 2. Methodology

The review reported according to Reporting Items for practice Guidelines in Healthcare (RIGHT) protocol. The systematic review was sent to clinical study registration.

### 2.1. Search Strategy

A systematic and exhaustive search of the literature was performed from Cochrane review, PubMed, Google Scholar, and Hinari databases. The search was performed using key words for PubMed, Cochrane, and Hinari (postoperative pain AND breast cancer OR pain AND mastectomy OR regional anesthesia AND breast cancer OR pain AND breast cancer), and full-sentence search was carried out for Google Scholar.

### 2.2. Inclusion and Exclusion Criteria

#### 2.2.1. Inclusion Criteria

Observational, interventional studies, guidelines, systematic review and meta-analysis, full articles published from 2009 to August, 2022, and articles written in English language were included in this review.

#### 2.2.2. Exclusion Criteria

Articles without relevant outcomes, postoperative pain prevention of other types of cancers, nonsurgical interventions of breast cancer, and reconstruction surgeries were excluded.

### 2.3. Data Extraction

**Figure 1:**
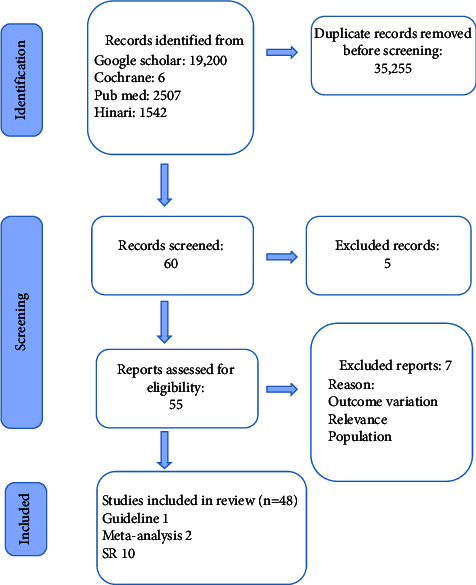
Preferred reporting items for systematic review and meta-analysis (PRISMA) flow diagram.

Systematical data extraction was made using the MeSH term and applying PICO (Supplementary Table 1a). Search engine results were filtered based on the interventions, outcome, population data, and methodological quality. The articles involving the prevention of postoperative pain in breast cancer surgical patients with relevant outcomes were selected. Extraction and filtering were carried out using a patient population and exclusion criteria's: 1 Guideline, 2 meta-analyses, 10 systematic reviews, 25 RCTs, and 10 observational studies were appraised for quality evidence (Figure 1), and Supplementary Table 1b.

The conclusion was made based on their level of evidence and grades of recommendations that were adapted from Oxford Center for Evidence-Based Medicine (Table 1).

## 3. Results

### 3.1. Pain Assessment

To identify the presence of pain and assess the efficacy of pain relief, pain assessment should be performed. Although according to a large body of research, there is significant interindividual heterogeneity in how people perceive standardized acute noxious stimulus, the widely accepted pain scoring methods are the visual analog scale and the numeric rating scale [14].

**Figure 2:**
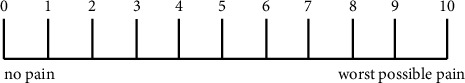
Visual analog scale and numeric rating scale.

The Visual Analogue Scale (VAS) is a simple tool, it has a high sensitivity for identifying treatment effects, and parametric tests can be used to analyze its outcomes. It is a continuous scale, made up of two verbal descriptions, such as “no pain” and “worst imaginable pain,” and a horizontal or vertical line that is typically 100 mm long [15] (Figure 2).

The Numeric Rating Scale (NRS) is a single, 11-point numerical scale that has been thoroughly verified for use with numerous patient types. NRS data are easily documented, comprehensible, and compliant with regulatory criteria for pain assessment and documentation. The pain scores are denoted by the following numbers: 0 = no pain, 1–3 = mild pain, 4–6 = moderate pain, and 7–10 = severe pain [15] (Figure 2).

### 3.2. Nonpharmacological Management of Postoperative Breast Cancer Pain

#### 3.2.1. Acupuncture

At the first visit of a study conducted by Jessica Quinlan et al., patients who were randomly assigned to the acupuncture group displayed statistically significant improvements in pain. Contrarily, patients who were randomly assigned to the control group did not exhibit any statistically significant variations in pain during the visit between pre- and poststandard care assessments. Postoperative acupuncture treatment (in addition to standard care) reduced pain, nausea, and anxiety while improving coping skills [16] **LOE** = **1c** and **GOR** **=** **B**.

#### 3.2.2. Aroma Therapy

Postoperative aromatherapy with lavender oil for 20 minutes is believed to decrease pain by decreasing anxiety and stress, but the RCTs included in the systematic review and meta-analysis did not get a significant relationship between postoperative pain and aromatherapy. Further interventional studies with a large sample size are required for strong recommendation [17] **LOE** = **1a** and **GOR** = **A**.

#### 3.2.3. Foot Massage

A foot massage (FM), characterized as the manual manipulation of soft tissues with the intention of improving the performance of various bodily systems, is when performed by a skilled therapist (nurses, physiotherapists, or trainees), an affordable and secure intervention on appropriately evaluated patients. The quasi-experimental study conducted in 2016 demonstrated that a 20-minute FM intervention dramatically decreased levels of postoperative discomfort within the first and second hours [18] **LOE** = **1c** and **GOR** **=** **B**.

#### 3.2.4. Physical Exercise

Exercise, acupuncture, cryotherapy, biofeedback, transcutaneous electrical nerve stimulation, and massage treatment are among the physical therapy methods that have been suggested by Kannan et al. for relieving postmastectomy pain. Exercise group, when compared to control, according to the meta-analysis, could be regarded as a vital part of quality of life and pain management for women with postmastectomy pain syndrome (PMPS) because it is a low cost and safe intervention [19] **LOE** = **1a** and **GOR** = **A**.

#### 3.2.5. Music

Perioperative music therapy, especially with the patient's preference significantly reduced postoperative pain in RCT's included in the review. Music was delivered 5 minutes before surgery; in most studies, it continued intraoperatively and twice a day postoperatively. This decreased patients anxiousness and stress in turn reducing their pain score in comparison with those who did not listen to music throughout [17] **LOE** = **1a** and **GOR** = **A**.

#### 3.2.6. Sleep

In the last two decades, a growing body of data has confirmed that there is an inverse link between pain and sleep. In a study conducted on 24 women scheduled for breast cancer surgery in New York, USA, it was discovered that poor sleep quality the night before surgery was linked to significantly higher pain intensity and pain interference with everyday activities the week after surgery [20] **LOE** = **2c**.

Women with poor sleep quality who undergone breast cancer surgery had a greater frequency of severe postoperative pain, reported higher NRS scores, and required more rescue analgesics in the first postoperative 24 hours which were statistically significant [21] **LOE** = **2b**. A prospective observational study conducted in 2021 found, significant effects between preoperative sleep disruptions in women and more intense peak pain after movement within the first 24 hours after surgery [22] **LOE** = **2c**, **GOR** **=** **B,** and **GOR** **=** **C**.

#### 3.2.7. Psychological

An RCT conducted in Buchan, Germany found that psychological techniques that focus on enhancing positive expectations are effective in enhancing patient-reported outcomes for postoperative pain management. This can be achieved with encouraging verbal suggestions combined with other tried-and-true components of doctor-patient communication, including improved empathy, trust, and an upbeat, caring attitude.

Postoperative pain ratings were lower in women who received positive suggestions compared with neutral verbal comments, irrespective of other interventions [23] **LOE** = **1b**. In addition to this, the assessment of coping strategies among breast cancer patients with preoperative pain did not have any predictive value for increased postoperative pain. But, those with greater isolated anxiety ratings for the expectations of higher postoperative pain predict more intense postoperative pain after breast cancer surgery [23–25] **LOE** = **1b**, **LOE** = **2c**, **GOR** = **A**, and **GOR** **=** **C**.

### 3.3. Pharmacological Management of Postoperative Breast Cancer Pain

#### 3.3.1. Nonsteroidal Anti-Inflammatory Drugs

In a systematic review conducted by Klifto KM regarding women having breast surgery and the use of nonsteroidal anti-inflammatory drugs (NSAIDs), the study discovered evidence that perioperative (preoperative and/or postoperative) NSAIDs may lessen pain severity as well as 30% decrement in opioid use within 24 hours after surgery. However, there was insufficient evidence to support the idea that perioperative NSAIDs would affect the likelihood of breast hematoma within 90 days of breast surgery (requiring reoperation, interventional drainage, or no treatment) and further strong, large-scale RCTs are needed before definite conclusions are made [10, 26] **LOE** = **1a**, **LOE** = **2a**, **GOR** = **A**, and **GOR** **=** **B**.

#### 3.3.2. Opioids

On postoperative day 1 of a prospective cohort study conducted in Washington, USA, the pain levels of opioid free anesthesia (OFA) patients were much lower. Now a day opioids are being replaced by other components of multimodal analgesia regimens and nerve blocks because of their undeniable side effects which has greater impact on the quality of life of the patients after surgery [27] **LOE** = **2a**.

In a continued opioid free anesthesia regimen, an RCT conducted by Gürkan in 2020 comparing paravertebral and erector spinae block with IV morphine found nearly identical decrement in morphine consumptions were found with both erector spinae and paravertebral block techniques. Such decreased morphine (opioid) consumption could make a clinically significant difference in patient care and opioid free patient's quality of life [28] **LOE** = **1b** and **GOR** = **A**.

#### 3.3.3. Anticonvulsant

*(1) Gabapentin*. Anticonvulsant medicine gabapentin is extensively and successfully used to treat chronic neuropathic pain and it has recently been discovered to be helpful for lowering acute postoperative pain when given prior to surgery. Without excessive sedation or other side effects, anticipatory administration of a single oral dosage of 600 mg of gabapentin resulted in a reduction in postoperative analgesic requirements as compared to placebo [29] **LOE** = **1b**. Patients in the gabapentin group had decreased pain scores at 30 minutes, 1 hour, and 2 hours postoperatively compared to placebo, and intraoperative propofol use was considerably lower [30, 31] **LOE** = **1b**, **1a**, and **GOR** = **A**.

*(2) Pregabalin*. A systematic review and meta-analysis consisting of twelve RCT, pregabalin, which has a higher bioavailability than gabapentin resulting in a superior pharmacokinetic profile, lessens pain during recovery and the need for opioids, but it does not lessen discomfort throughout 24 hours [32] **LOE** = **1a** and **GOR** = **A**.

*(3) Deluxetine*. Duloxetine (a serotonin norepinephrine reuptake inhibitor) has lately been employed as a component of multimodal analgesia in perioperative settings, according to current studies on the effects of perioperative use for treating postoperative pain and opioid intake. The ideal dose for patients undergoing modified radical mastectomy prior to surgery is 60 mg of oral duloxetine, unlike the 90 mg which was associated with an increased level of sedation up to 8 hours postoperatively [33] **LOE** = **1b** and **GOR** = **A**.

*(4) Gabapentin vs. Pregabalin*. Pregabalin and gabapentin appear to lessen the use of opioids in the recovery area. Pregabalin does not lessen pain 24 hours after breast cancer surgery; gabapentin does. Neither medication influences the emergence of persistent postsurgical pain [31, 32] **LOE** = **1a** and **GOR** = **A**.

*(5) Pregabalin vs. Ketamine*. In a study conducted in Egypt comparing pregabalin with ketamine With a *P* value of 0.001, it was discovered that preoperative oral 150 mg pregabalin or 0.5 mg/kg ketamine use lowers overall postoperative morphine consumption. The need for opioids was the same in the pregabalin and ketamine groups [34] **LOE** = **1b** and **GOR** = **A**.

#### 3.3.4. Ketamine

Due to its side effects ketamine's, an N-methyl-D-aspartate (NMDA) receptor antagonist, use was formerly restricted. However, a few years ago, this medicine was rescued, and fresh lines of research have shown that it has good analgesic power. In a retrospective cohort study conducted in 2021 comparing ketamine and opioid, the ketamine group had a significantly reduced incidence of acute postoperative pain than the opioid group, as well as a significantly lower pain intensity [35] **LOE** = **2b**.

The available data of meta-analysis conducted in China demonstrated that both intravenous ketamine administration and ketamine added to bupivacaine in paravertebral blocks effectively reduces the cumulative consumption of morphine in patients undergoing breast surgery while reducing the risk of either gastrointestinal or CNS adverse events [36] **LOE** = **1b**, **GOR** = **A**, and **GOR** = **B**.

#### 3.3.5. Dexamethasone

RCT conducted by Gómez-Hernández et al. with a sample size of 70 breast cancer patients found that preoperative dexamethasone at 8 mg alleviates nausea, vomiting, pain, and decreases the need for analgesics and antiemetics in women right after breast cancer surgery without any discernible side effects [37] **LOE** = **1b**. RCT's conducted in Mexico and Denmark also supports this finding of using 8 mg of dexamethasone to relief acute postoperative pain rather than adding the dosage [38, 39] **LOE** = **1b**; **GOR** = **A**.

#### 3.3.6. Intravenous Lidocaine

Despite claims of analgesic advantages for neuropathic pain of abdominal and thoracic procedures, intravenous lidocaine appears to provide when used for breasts, has little benefit for analgesia operation. In one RCT of patients for mastectomy, there was no appreciable difference between patients who received an intraoperative infusion of intravenous lidocaine (3 mg/kg) and those in the placebo group in terms of pain levels or postoperative analgesic use. Further investigation is needed for the conclusion of this finding [10] **LOE** = **1a**; **GOR** = **A**.

#### 3.3.7. Local Anesthesia Wound Infiltration

For LA wound infiltrations, the most used solutions are ropivacaine, bupivacaine, and lidocaine, which results in a reduction in pain scores and reduced rescue opioid consumption. Although the effect may never last for more than 24 hours and it is most commonly limited up to six hour, the use should be considered in patients with minor to moderate invasive surgeries like lumpectomy and partial lumpectomy since the PPP from these procedures is mild to moderate and the intensity decreases over time [9] **LOE** = **1a** and **GOR** = **A**.

#### 3.3.8. Local Anesthesia Wound Infusion

Postoperatively, local anesthesia can be continuously infused by inserting a catheter directly into the wound. Uncertainty exists regarding the analgesic benefits that these catheters offer following breast surgery. Wound catheter infusions with local anesthetic for breast cancer procedures may not provide much in the way of therapeutic benefits, but the subject warrants more research [10] **LOE** = **1a** and **GOR** = **A**.

#### 3.3.9. Lidocaine and Magnesium

In an RCT comparing the effect of IV lidocaine (3 mg/kg) and magnesium (50 mg/kg) there was synergistic analgesic effect that was seen in patients who took both lidocaine and magnesium sulfate, both intraoperative and postoperative reduced pain scores throughout the study, and decrement in opioid consumption was observed. This is due to theory that the combined effect of blocking sodium channels and more strongly blocking NMDA receptors should have a more noticeable impact on the sensory system of glutamatergic synapses, resulting in a coordinated synergism. It can serve as effective multimodal component [40] **LOE** = **1b** and **GOR** = **A**.

#### 3.3.10. Intraoperative Esmolol

Women who received intravenous esmolol (0.5 mg/kg) as an adjuvant in a randomized trial study for mastectomy reported much less postoperative pain than those who got a placebo (saline). Esmolol-treated women consumed less morphine and metamizole than placebo-treated women. This finding shows that esmolol, a beta-blocker, is associated with enhanced cardiovascular stability and may significantly minimize the need for intraoperative anesthetics during mastectomy. Further uniform interventions should be conducted to strengthen the result of the study and the recommendation [41] **LOE** = **1b** and **GOR** = **A**.

### 3.4. Regional Anesthesia

Peripheral nerve blocks are become a popular and crucial part of multimodal analgesia as a means of preventing and treating postoperative pain since the sensory nerves of the thoracic wall are primarily responsible for most of the pain experienced during breast surgery.

The outcomes analyzed by a systematic review and meta-analysis consisting of 79 RCT showed that paravertebral blocks consistently provided the best results, but they also had a higher risk of complications, and SAPB was assessed to have a high likelihood of decreasing 24-hour resting pain [42]. **LOE** = **1a** and **GOR** = **A**.

#### 3.4.1. Paravertebral Nerve Blocks

According to a retrospective cohort studies conducted in Japan and India, thoracic paravertebral block combined with general anesthesia decreased the incidence of postoperative chronic pain for more than a year following breast cancer surgery and postoperative acute pain within the first few hours following surgery [43, 44] **LOE** = **2b** and **LOE** = **2b**.

A systematic review conducted by Chhabra associated the paravertebral block (PVB) technique with reduced postoperative pain during rest and on motion from 6 to 24 hours, but as the outcomes were assessed by a small number of studies, more large scaled multicentered studies with uniform design are needed to conclude these outcomes. All complications of the paravertebral technique, such as Horner's syndrome, epidural spread of the local anesthetics, bloody tap, or pneumothorax due to pleural injection, are mostly self-limited and does not need any treatment [45] **LOE** = **1a**.

A multicenter RCT conducted by Albi-Feldzer to validate the long term effect of PVB on chronic postoperative pain comparing it with saline injection in to the paravertebral space which found no significant difference between the two effect on the long run of postoperative pain management [46] **LOE** = **1b**. Because of the amazingly low incidence of postoperative pain, it was not possible to rich conclusion in comparing the efficacy of both PVB with local anesthetics and saline [47] **LOE** = **1b**, **GOR** = **A**, and **GOR** **=** **B**.

#### 3.4.2. Paravertebral Blocks: Extended Duration

When added to a long-acting local anesthetic, clonidine (75 mg) can extend the analgesic block effect for up to 72 hours following administration, similar to how adding fentanyl to a local anesthetic for paravertebral block enhances analgesia for breast surgeries. Despite the fact that there is conflicting evidence about the effects of continuous paravertebral blocks versus single-injection blocks using a continuous paravertebral block is a more reliable technique to extend block duration for more than 12–16 hours after a single-injection paravertebral block can produce analgesia for up to 72 hours according to systematic review of RCTs [10] **LOE** = **1a** and **GOR** = **A**.

#### 3.4.3. Paravertebral Blocks with TIVA

Total intravenous anesthesia (TIVA), together with paravertebral blocks may boost the benefits of a regional anesthetic technique. Increased postoperative analgesia, reduced opioid needs and reduced recovery room stays have been mentioned in multiple RCTs included in the systematic review of Cheng and Ilfeld [10] **LOE** = **1a**. For major breast surgeries like a mastectomy with or without axillary node dissection, paravertebral block plus TIVA should be taken into consideration.

Studies showed that this intervention, when compared to general anesthesia alone, was associated with lower postoperative pain scores, lower systemic analgesia consumption, and a shorter length of hospital stay was noted with addition of TIVA. The effectiveness of this technique for postdischarge pain needs additional multicentered interventional studies. Reference [9] **LOE** = **1a** and **GOR** = **A**.

#### 3.4.4. Brachial Plexus Blocks

Depending on the amount of local anesthetic used, the interscalene block offers a sensory block in the brachial plexus and T1-T2 distribution. However, in an RCT conducted to assess the effectiveness of interscalene brachial plexus block, it could not block the intercostal nerves innervating the skin, and patients needed systemic opioid analgesics in addition to the routine nonsteroid anti-inflammatory analgesics use to provide postoperative analgesia [48] **LOE** = **1b**. The results of another RCT included in a systematic review is convincing and shows that a single interscalene block injection may provide analgesic benefits following breast surgery but needs further investigation on a large scale studies [10] **LOE** = **1b** and **GOR** = **A**.

#### 3.4.5. Thoracic Epidural Analgesia

Thoracic epidural block after major breast oncologic surgery provides comparable postoperative analgesia when compared with the current data on other available technique, even though there is no direct comparison that currently exists between thoracic and others including continuous paravertebral blocks. Technique-specific limitations include the obligation to stay in the hospital until the epidural is removed, the cautious use of anticoagulants, and the hypotension induced by the sympathectomy [10] **LOE** = **1a**.

An RCT comparing thoracic epidural with thoracic paravertebral in terms of VAS, there was no statistically significant difference between the examined groups, indicating that the two regional techniques analgesic profiles were comparable in both groups. However, the study group who took thoracic epidural showed instability regarding intraoperative hemodynamic in both HR and BP [49] **LOE** = **1b** and **GOR** = **A**.

#### 3.4.6. Cervical Epidural Analgesia

LA infusion (with or without opioid) into the cervical epidural area is a more cephalic epidural alternative. For major breast surgery, this local method may be employed to administer both intraoperative anesthetic and postoperative analgesia. The catheter insertion made between the C7 and T1 vertebrae would provide analgesic benefit because the brachial plexus (C5–C8) is the source of the pectoralis muscle's innervation.

This is also supported by a previous study that found that a cervical epidural block provides a better sensory block for thoracic procedures than a high thoracic epidural block; however, there are no RCTs that validate or evaluate this strategy in relation to other analgesic methods. Due to their nature, case studies and short patient series that describe the use of this approach cannot accurately assess the procedure's risks. Further, strong interventional studies on a large scale base should be implemented for the standardization of this technique [10] **LOE** = **1a** and **GOR** = **A**.

#### 3.4.7. Interfacial Plane Blocks (PECs I and PECs II)

Thoracic interfascial plane blocks are considered safe and easy superficial nerve blocks for oncologic breast surgery. The medial cutaneous nerve of the arm and forearm, intercosto-brachial, lateral cutaneous branch, long thoracic, and thoracodorsal nerves can all be anesthetized by blocking the pectoral nerves (PECS), which has analgesic effects on the lateral mammary region [50]. A double-blind randomized trial in France found when postoperative analgesia is adjusted using dexamethasone, wound infiltration with a long-acting LA, acetaminophen, and NSAIDs with/without morphine, using a PECs I block may not be necessary to reduce the pain scores for patients having breast cancer surgery [51] **LOE** = **1b**.

An RCT conducted by Neethu et al. and a meta-analysis conducted by Danielle Lovett both shared common conclusion that, with less restriction to shoulder movement (pain-free mobilization) on the surgical site up to 4 hours and 5 hours after surgery, combined PECS I and PECS II block in adult women undergoing modified radical mastectomy effectively reduces the overall amount of fentanyl required in the intraoperative/postoperative period (up to 24 hours) [52, 53] **LOE** = **1a** and **LOE** = **1b**.

Another randomized trial from Nigata, Japan, that evaluated the effects of PECS block in combination with general anesthesia on postoperative pain measured by NRS and intraoperative doses of propofol and remifentanil concluded that, PECS block significantly decreased propofol requirement but not that for remifentanil requirement during surgery and POD1. This is due to the blocks adequacy in blocking the lateral cutaneous branches of spinal nerves at the level of T2 to T6, and with a sufficient dose the anterior cutaneous branches might be blocked as the LA may penetrate the external intercostal muscles. If the LA is inadequate, the medial part of the chest wall will not be pain free and the analgesia will be inadequate needing additional systemic analgesia [54] **LOE** = **1b** and **GOR** = **A**.

#### 3.4.8. Erector Spinae Block

Injection of local anesthetics within the erector spinae plane is a relatively easy and secure block for breast surgery procedures that eases pain and help decrease opioid consumption. In an RCT conducted in 2022 with a control group taking general anesthesia in the traditional way, at the 2^nd^ and 4^th^ hour postoperatively, the severity of acute postoperative pain was noticeably greater in the control group than in the ESP group. In addition, patients' and assessor's ratings of treatment satisfaction in the ESP group were higher than those in the control group [55] **LOE** = **1b** and **GOR** = **A**.

#### 3.4.9. PECS Block vs. Erector Spinae Block

Patients who underwent breast cancer surgery experienced stronger analgesic efficacy from PECS II block and ESP block than from systemic analgesia according to a meta-analysis conducted in 2020 comparing PECS and erector spinae block. Compared to systemic analgesia, PECS II block dramatically reduced opioid use and pain score. While pain scores in patients receiving ESP block did not increase not very different from those with systemic analgesia [56] **LOE** = **1a**. An RCT conducted in Turkey by Başak Altıparmak had similar finding while comparing it in regard with tramadol consumption following radical mastectomy surgery, modified PECS block significantly decreased postoperative tramadol use and pain scores compared to ESP block [57] **LOE** = **1b** and **GOR** = **A**.

#### 3.4.10. Serratus Anterior Plane Block

A novel fascial plane block technique is the SAP block. The anterior and lateral chest walls respond well to analgesia when the second to ninth intercostal nerves (T2–T9) lateral cutaneous branch is blocked. The SAP block methodology is simpler and less fraught with danger than other regional block methods. According to the study's findings, preoperative SAP block can significantly reduce the amount of perioperative opioids consumed during a modified radical mastectomy for breast cancer, as well as the perioperative surgical stress response, perioperative patient satisfaction with postoperative pain management, and the incidence of PMPS. All of these factors are favorable to surgery [58] **LOE** = **1b** and **GOR** = **A**.

#### 3.4.11. PECS II vs. Serratus Anterior Block

The PECS 2 block decrease's the rate of moderate to severe PPP after six months of oncologic breast surgeries compared with the serratus plane block [59] **LOE** = **1b**. Given that the risk-benefit ratio favors PECs block, it should be used as the first-line regional anesthetic method in a multimodal analgesic regimen during breast surgery [60] **LOE** = **1a** and **GOR** = **A**.

#### 3.4.12. PECS Block vs. Paravertebral Block

A systematic review and meta-analysis with a trial sequential analysis stated that there is no strong evidence that suggests the superiority of PECS block over paravertebral block for breast surgeries [61] **LOE** = **1a** and **GOR** = **A**.

## 4. Conclusion

Postoperative pain is subdivided into acute and chronic. While, acute postoperative pain includes the time immediately after surgery to 3 months postoperatively either at rest or during movement, a postoperative pain is a pain that occurs after 3 months depending on the surgeries involvement and the management of acute PPP. Age, anxiety, depression, pain and genetics may play important role as patient factors while axillary staging and lymph node dissection can be intraoperative factors for developing chronic PPP.

**Figure 3:**
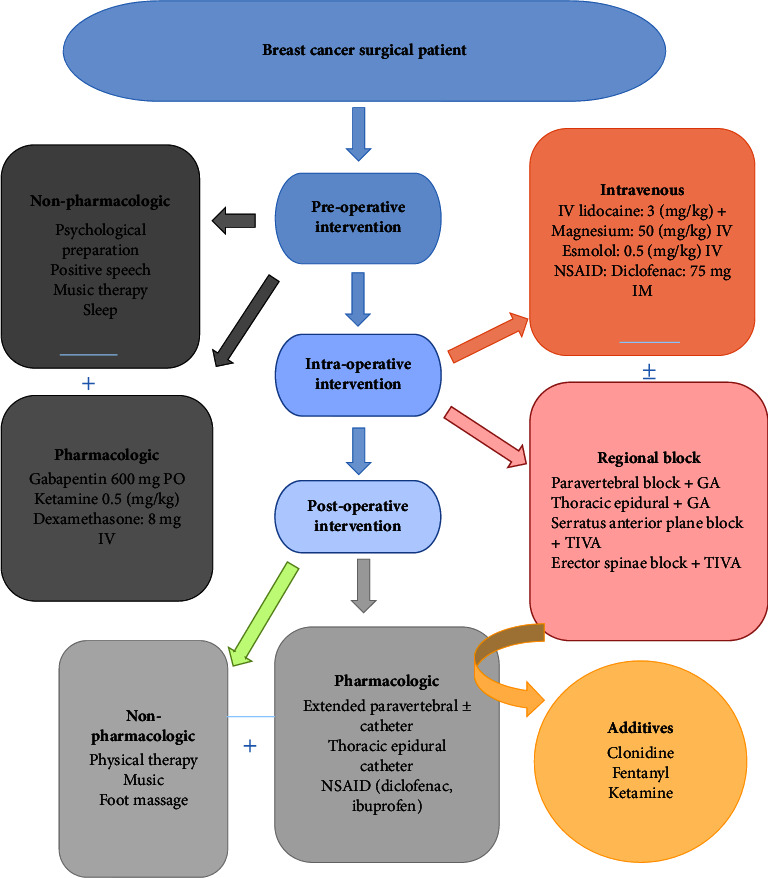
Management flow chart for perioperative prevention and management of pain for breast cancer surgical patients.

We recommend perioperative use of a nonpharmacological approach (including preoperative positive inspirational words and positive expectation) as adjunct to intraoperative regional anesthetic technique with general anesthesia preferably TIVA with calculated appropriate dose of standard pharmacological multimodal regimens. Even though preoperative adequate sleep does not have strong evidence for firm recommendation as an effective nonpharmacological method to reduce postoperative pain, we suggest it with low grade recommendation **LOE** = **2c** and **GOR** = **C**. For postoperative analgesia, an extended form of intraoperative regional technique, nonpharmacologic technique, and NSAIDs can be used with the opioid-sparing anesthesia technique **LOE** = **1a**, **1b GOR** = **A,** and **GOR** **=** **B** (Figure 3). The summary of recommendations was presented with their strength of evidence (Table 2).

## Figures and Tables

**Table 1:** Level of evidence and grades of recommendation [13].

Level of evidence	Grading criteria	Grade of recommendation
1a	Systematic reviews of RCTs including meta-analysis	A
1b	RCT with narrow confidence interval	A
1c	All or none randomized controlled trials	B
2a	Systematic review of cohort study	B
2b	Cohort including low quality RCT	C
2c	Outcome research study	C
3a	Systematic review of case control studies	C
3b	Case control study	C
4	Case series, poor quality cohort and case control studies	C
5	Expert opinion without explicit critical appraisal, or based on physiology, bench research or “first principles”	D

**Table 2:** The summary of recommendation and strength of evidence for perioperative pain management in breast cancer patients.

S. No.	Pain management methods			Grades of recommendation	Strength of evidence
*1*	*Preoperative pain management approaches*				
	Nonpharmacologic methods	Psychological preparation		A	1b
		Adequate sleep		B	2b
		Music therapy		A	1a
	Pharmacologic methods; systemic analgesia	Anticonvulsant	Gabapentin	A	1a
			Deluxetine	A	1b
			Pregabalin	A	1a
		Ketamine		A	1b
		Dexamethasone 8 mg		A	1b

*2*	*Intraoperative pain management using the pharmacological methods*				
	Systemic analgesia	IV lidocaine 3 mg/kg with magnesium 50 mg/kg		A	1b
		Esmolol		A	1b
		Local anesthesia wound infiltration with ropivacaine or bupivacaine		A	1a
		NSAIDs (diclofenac or ibuprofen)		A	1a
		Opioid free anesthesia		A	1b
	Regional technique	Paravertebral extended block (with clonidine, fentanyl, or ketamine) + TIVA		A	1a
		Combined PECS I and PECS II block + TIVA		A	1a
		Thoracic epidural anesthesia + TIVA		A	1a, 1b
		Serratus anterior plane block + MMA + TIVA		A	1a
		Erector spinae block + MMA + TIVAs		A	1b

*3*	*Postoperative pain management*				
	Nonpharmacologic methods	Acupuncture		B	1c
		Physical exercise		A	1a
		Music therapy		A	1a
		Foot massage		B	1c
	Pharmacologic interventions	Extended paravertebral (with or without catheter)		A	1a
		Thoracic epidural catheter		A	1a
		Additives: Clonidine, fentanyl, or ketamine		A	1b
		NSAIDs		A	1a

## Data Availability

The data used to support the findings of this study are made available from the corresponding author upon reasonable request.

## Abbreviations

BC: Breast cancer
GOE: Grade of evidence
LOE: Level of evidence
PMPS: Post mastectomy pain syndrome
RCT: Randomized control trial
RIGHT: Reporting Items for Practice Guidelines in Healthcare Protocol.

## Supplementary Materials

Supplementary Materials Supplementary Table 1a describes systematic data extraction by using PICO; and Supplementary Table 1b describes the studies included in the review for the guideline.
