# Supplementary material for: Evidence-Based Guideline on the Prevention and Management of Perioperative Pain for Breast Cancer Peoples in a Low-Resource Setting: A Systematic Review Article
Source: Anesthesiol Res Pract. 2023 Nov 3;2023:5668399. doi: 10.1155/2023/5668399 (PMC10637850; doi:10.1155/2023/5668399)
Supplement: Supplementary Materials — Supplementary Table 1a describes systematic data extraction by using PICO; and Supplementary Table 1b describes the studies included in the review for the guideline. [file 5668399.f1.zip › Supplementary table - 1a (1).docx]

Supplementary table – 1a. Data extraction; Population, Intervention, Comparator, Outcome (PICO), and MeSH term

| PICO | Key terms | Results | | | |
| --- | --- | --- | --- | --- | --- |
| **Target population:**  Breast cancer surgical patients  Surgeons and anesthesia providers  PACU team and other health providers  Patients families | Pain AND mastectomy | Google scholar | PubMed | Hinari | Result |
|  |  |  | 2,507 articles | 6 articles | P=16;  2 OS  10RCT  4 SR  C=2;  1 MA  1 RCT |
| Intervention  Perioperative non-pharmacological pain management  Peri-operative pharmacologic pain management | Anesthesia AND breast cancer | 12,000 articles |  |  | 8;  8 OS |
| Comparison:  GA alone vs regional + GA  Between regional anesthesia techniques with vs without non-pharmacologic intervention | Regional anesthesia AND mastectomy | 19,200 articles |  |  | 15;  13 RCT  2 SR |
| Outcome:  Reducing pre-operative anxiety and stress  Reduce intraoperative pain  Reduce acute post-operative pain  Reduce chronic post-operative pain | Post-operative pain AND mastectomy |  |  | 1542 articles | 7;  1 G  1 MA  1 RCT  4 SR |

Note. P= PubMed, C= Cochrane, MA= Meta-analysis, SR= Systematic review, G= Guideline, RCT= Randomized controlled trial, OS= Observational studies.
