# Supplementary material for: Evidence-Based Guideline on the Prevention and Management of Perioperative Pain for Breast Cancer Peoples in a Low-Resource Setting: A Systematic Review Article
Source: Anesthesiol Res Pract. 2023 Nov 3;2023:5668399. doi: 10.1155/2023/5668399 (PMC10637850; doi:10.1155/2023/5668399)
Supplement: Supplementary Materials — Supplementary Table 1a describes systematic data extraction by using PICO; and Supplementary Table 1b describes the studies included in the review for the guideline. [file 5668399.f1.zip › Supplementary table -1b.docx]

Supplementary table - 1b. The studies included in the review for the guideline

| **No** | **Author/year** | **Study** | **Population** | **Sample size/design** | **Finding of the evidence** | **LOE** |
| --- | --- | --- | --- | --- | --- | --- |
| 1 | Safaa M et al/2022 | Comparative study between thoracic epidural and  ultrasound-guided thoracic paravertebral block in perioperative  pain management for mastectomy | Breast cancer surgical patients | 60, RCT | Ultrasound‑guided PVB is an effective technique showing greater hemo-dynamics stability and pain control compared with epidural analgesia for mastectomy | 1b |
| 2 | Caroline A. Kinget al/2020 | Opioid-free anesthesia for patients undergoing mastectomy | Breast cancer surgical patients | 48, matched cohort study | This study proved that OFA is more effective than a conventional opioid-based regimen at reducing post-operative pain and nausea. Therefore, OFA is viable in people who have had mastectomy and should be further assessed in a few patients. | 2b |
| 3 | Yavuz Gürkanet al/2020 | Erector spinae plane block and thoracic paravertebral block for breast  surgery compared to IV-morphine | Breast cancer surgical patients | 75, RCT | This study has shown that ESP block and PVB provided adequate analgesia in patients  undergoing breast surgery and have an opioid sparing effect by reducing morphine consumption | 1b |
| 4 | etunde Oluwafunmilayo Tola et al/2021 | Effects of non- pharmacological interventions on pre-operative anxiety and postoperative pain in patients undergoing breast cancer surgery | Breast cancer surgical patients | 6 RCT, systematic review | Music, aromatherapy and acupuncture appeared to be effective for reducing pre-operative anxiety and postoperative pain in women undergoing breast cancer surgery | 1a |
| 5 | J bruce et al, 2017 | Chronic preoperative pain and psychological robustness predict  acute postoperative pain outcomes after surgery for breast cancer | Breast cancer surgical patients | 405, prospective cohort | Chronic preoperative pain, axillary surgery and psychological robustness significantly predicted acute pain outcomes after surgery for breast cancer | 2b |
| 6 | Priya Kannan et al/2022 | Efficacy of physical therapy interventions on quality of life and upper quadrant pain severity in women with post‑ mastectomy pain  syndrome | Breast cancer surgical patients | 18 RCT, systematic review and meta-analysis | The meta-analyses found that exercise is beneficial for improving the quality of life and pain severity of women with post mastectomy pain syndrome | 1a |
| 7 | Chhabra A et al/ 2021 | Paravertebral anesthesia with or without sedation versus general  anesthesia for women undergoing breast cancer surgery | Breast cancer surgical patients | 9 RCT (n=614), systematic review | Paravertebral anesthesia may reduce the 24-hour postoperative analgesic requirement compared to general anesthesia | 1a |
| 8 | Maria Luiza Alves et al/2013 | Preoperative coping mechanisms have no predictive value  for postoperative pain in breast cancer | Breast cancer surgical patients | 139, prospective observational | Coping mechanisms  and pain in the preoperative period did not have a strong predictive value for additional post-operative pain, but those with a higher anxiety score had greater pain. | 2c |
| 9 | Caroline E et al/ 2009 | Disrupted Sleep the Night Before Breast  Surgery Is Associated with Increased  Postoperative Pain | Breast cancer surgical patients | 24, prospective observational | disrupted sleep the night before surgery was related to signiﬁcantly greater pain severity, and to pain interference with daily activities over the week after surgery | 2c |
| 10 | A.O. Cortés-Flores MD, 2017 | Effects of pre-operative dexamethasone on post-operative  pain, nausea, vomiting and respiratory function in women  undergoing conservative breast surgery for cancer | Breast cancer surgical patients | 80, RCT | a single dose of dexamethasone should be included in the multimodal therapy offered to patients being treated surgically for malignant breast disease under general  anesthesia | 1b |
| 11 | Jorge Gómez-Hernández et al/ 2010 | Preoperative dexamethasone reduces Post-operative pain, nausea and vomiting  following mastectomy for breast cancer | Breast cancer surgical patients | 70, RCT | Preoperative intra-venous dexamethasone (8mg) can significantly reduce the incidence of PONV and pain in patients undergoing mastectomy with axillary dissection for breast cancer | 1b |
| 12 | Salwa Hagag Hussien Abdelaziz et al/ 2014 | Effect of foot massage on post-operative pain and vital signs in breast cancer | Breast cancer surgical patients | 60, quasi experimental | Foot massage is an effective modality in helping to relieve postoperative pain among women who have been treated with surgery for breast surgery. | 1c |
| 13 | Reetta M. Sipil et al, 2017 | Does expecting more pain make it more intense?  Factors associated with the first week pain  trajectories after breast cancer surgery | Breast cancer surgical patients | 563, prospective observational | Psychological distress, pain expectations, and patients’ reports of preoperative pain in the area to be  operated on should be recognized and assessed before surgery and need more efficient analgesic approach. | 2c |
| 14 | Neerja Bharti et al, 2013 | Effect of gabapentin pre-treatment on propofol consumption, hemodynamic  variables, and post-operative pain relief in breast cancer surgery | Breast cancer surgical patients | 40, RCT | Preoperative administration of gabapentin reduced intraoperative propofol requirements and  postoperative analgesic consumption in breast cancer patients undergoing total mastectomy | 1b |
| 15 | A. Jacobs et al/ 2020 | PROSPECT guideline for oncological breast surgery: a systematic review and procedure-speciﬁc postoperative  pain management recommendations | Breast cancer surgical patients | 53 RCT, 9 meta-analysis, PROSPECT guideline | the review has identiﬁed analgesic  regimens for optimal pain management after breast surgery. And also identiﬁed analgesic  interventions that are not recommended for pain  management in breast surgery | 1a |
| 16 | Mirian L ´opez et al/ 2021 | Prevention of Acute Postoperative Pain in Breast Cancer: A  Comparison between Opioids versus Ketamine in the  Intraoperatory Analgesia | Breast cancer surgical patients | 71, retrospective cohort | Administration of ketamine is more eﬀective than opioid use for acute postoperative pain prevention in breast cancer surgery because the ketamine  group presented with less pain than the opioid group at all measured times | 2b |
| 17 | Michelle Chiu et al/2014 | Reducing Persistent Postoperative Pain and Disability 1 Year  After Breast Cancer Surgery: Comparing Thoracic Paravertebral Block to Local Anesthetic  Inﬁltration | Breast cancer surgical patients | 145, RCT | The study reports a low incidence of chronic  pain 1 year following major breast cancer surgery | 2b |
| 18 | Rachel Aufforth et al/ 2012 | Paravertebral Blocks in Breast Cancer Surgery: Is There  a Difference in Postoperative Pain, Nausea, and Vomiting? | Breast cancer surgical patients | 337, observational | Patients undergoing breast cancer surgery  Who have para-vertebral blocks have similar postoperative  nausea and vomiting and similar post-operative pain scores  compared with patients without para-vertebral blocks. | 2c |
| 19 | Ichikawa Yuki et al, 2017 | PECS Block Provides Effective Post-operative  Pain Management for Breast Cancer  Surgery | Breast cancer surgical patients | 254, case control | The PECS block  provides effective postoperative analgesia within the first 24 hours after breast cancer surgery | 3b |
| 20 | Jérôme Cros et al/ 2018 | Pectoral I Block Does Not Improve Postoperative  Analgesia After Breast Cancer Surgery | Breast cancer surgical patients | 128, RCT | Peri-operative opioid consumption in the PACU and pain scores up to  24 hours after surgery did not differ between the patients who received Pecs I block with bupivacaine or saline. | 1b |
| 21 | Yoshinori Kamiya et al/ 2017 | Impact of pectoral nerve block on postoperative pain  and quality of recovery in patients undergoing breast  cancer surgery | Breast cancer surgical patients | 60, RCT | PECS block combined with general anesthesia  reduced the requirement for propofol but not that for remifentanil,  due to the inability of the PECS block to reach the internal mammary area | 1b |
| 22 | Marcin Wiech et al/ 2022 | The quality of recovery after erector spinae  plane block in patients undergoing breast  surgery | Breast cancer surgical patients | 196, retrospective cohort | To conclude, the results presented in our study showed the  superiority of the ESP block over a CON group in the quality of recovery in patients undergoing breast surgery. Moreover, the ESP block lessened pain severity and reduced opioid consumption. | 1b |
| 23 | Yaodan Bi et al/2020 | The Effect of Ketamine on Acute and Chronic Wound Pain in Patients Undergoing Breast Surgery | Breast cancer surgical patients | 13 RCT, systematic review and meta-analysis | Ketamine is an effective and safe multimodal analgesic in patients undergoing breast surgery, administered both intravenously and when added to bupivacaine in paravertebral blocks. Also showed a long-term benefit for preventing post-operative depression and postmastectomy pain syndrome. | 1a |
| 24 | Essam Mahran et al/ 2015 | Comparison of pregabalin versus ketamine in postoperative pain management in breast  cancer surgery | Breast cancer surgical patients | 90, RCT | Neither the use of preoperative IV ketamine 0.5 mg/kg nor the preoperative oral use of 150 mg pregabalin could reduce VAS scores,  but they were proven in this study to reduce postoperative opioid requirements rendering them to be a good multi-modal analgesia | 1b |
| 25 | Ajit S Rai MD et al/ 2017 | Preoperative pregabalin or gabapentin for postoperative acute and chronic pain  among patients undergoing breast cancer surgery | Breast cancer surgical patients | 12 RCT, Systematic review and meta-analysis of RCT | gabapentin may reduce acute post-operative pain and  opioid consumption within the first 24 hours. Pregabalin may have an effect in reducing pain in the recovery room, but not at 24-hours. | 1a |
| 26 | Sven Benson et al/ 2019 | Can a brief psychological expectancy intervention  improve postoperative pain? | Breast cancer surgical patients | 96, RCT | Psychological strategies targeting positive expectations are promising tools to improve patient-reported outcomes in postoperative pain management. | 1b |
| 27 | Mens¸ure Kaya et al, 2013 | Postoperative analgesia after modiﬁed radical mastectomy:  the efﬁcacy of interscalene brachial plexus block | Breast cancer surgical patients | 60, RCT | The study demonstrated that the use of interscalene block in patients undergoing MRM improved pain scores and reduced opioid conception during the first 24 hours. | 1b |
| 28 | loria S Cheng et al/ 2016 | A review of postoperative analgesia for  breast cancer surgery | Breast cancer surgical patients | 284 articles, literature review | paravertebral blocks have been associated with decreasing the development of persistent post-surgical pain | 2a |
| 29 | Hiroki Shimizu et al/2015 | Thoracic para-vertebral block reduced the incidence of chronic post-operative pain for more than 1 year after breast cancer surgery | Breast cancer surgical patients | 46, retrospective cohort | These results indicate that Thoracic Paravertebral block has the potential to reduce chronic pain for more than 1 year  after breast cancer surgery | 2b |
| 30 | Binggao Chai et al/ 2019 | Poor preoperative sleep quality is a risk factor for severe postoperative pain after breast cancer surgery | Breast cancer surgical patients | 108, prospective cohort | breast cancer patients with poor pre-operative sleep quality reported more severe post-operative pain, required more analgesics, experienced more complications, and had longer hospital stay. | 2b |
| 31 | Tuomo J. Meretoja et al/ 2022 | Comparison of Postoperative Pain in 70 Women with Breast Cancer Following General Anesthesia for Mastectomy with and without Serratus Anterior Plane Nerve Block | Breast cancer surgical patients | 70, RCT | preoperative SAP block can significantly reduce postoperative pain after modified radical mastectomy for breast cancer | 1b |
| 32 | Zhi-Wen Yao /2021 | Relationships of sleep disturbance, intestinal microbiota,  and postoperative pain in breast cancer patients | Breast cancer surgical patients | 36, Prospective observational | These findings suggest that the changed gut microbiota may be involved in sleep-pain interaction and could be  applied as a potential preventive method for postoperative pain | 2c |
| 33 | S.R. Humble et al/ 2012 | Therapeutic interventions to reduce  acute and chronic post-surgical pain after amputation,  thoracotomy or mastectomy | Breast cancer, thoracic and amputation surgical patients | 32 RCT, systematic review | Appropriate dose regimes of gaba-pentinoids, antidepressants, local anesthetics and regional anesthesia may potentially reduce the severity of both acute and chronic pain for patients. | 1a |
| 34 | Fabrı´cio T et al/ 2021 | Intra-operative esmolol and pain following  Mastectomy | Breast cancer surgical patients | 70, RCT | Esmolol was well tolerated, allowed a notable reduction in the dose of rescue analgesics and demonstrated superior efficacy compared to placebo for pain management after mastectomy. | 1b |
| 35 | Aline Albi-Feldzer et al/ 2021 | Preoperative Paravertebral  Block and Chronic Pain after Breast Cancer Surgery | Breast cancer surgical patients | 380, RCT | Paravertebral block did not reduce the incidence of chronic pain after breast surgery. Paravertebral block did result in less immediate post-operative pain | 1b |
| 36 | Mendonça, Fabricio T/ 2020 | Synergistic effect of the association between lidocaine and magnesium sulfate on peri-operative pain after mastectomy | Breast cancer surgical patients | 120, RCT | There is a synergistic effect of the use of both lidocaine and magnesium in peri-operative pain. This may be another potential strategy in the multimodal  analgesia regimen. | 1b |
| 37 | Diab Fuad Hetta et al/ 2020 | Pre-operative duloxetine to improve acute pain and quality of recovery in  patients undergoing modified radical mastectomy | Breast cancer surgical patients | 88, RCT | Preoperative oral Duloxetine of 60 mg, for patients subjected to MRM is the optimal dose considering its analgesic efficacy and side effects. | 1b |
| 38 | V K Grover et al/ 2010 | A single dose of preoperative gabapentin for pain reduction and requirement of morphine after total mastectomy and axillary dissection | Breast cancer surgical patients | 50, RCT | A single low dose of 600 mg gabapentin administered 1 h prior to surgery produced effective and significant postoperative analgesia after total mastectomy and axillary dissection without significant side effects. | 1b |
| 39 | Kristin Julia Steinthorsdottir et al/ 2020 | Dexamethasone Dose and Early Postoperative Recovery after Mastectomy | Breast cancer surgical patients | 130, RCT | No evidence was found of 24 mg versus 8 mg of dexamethasone affecting the primary outcome regarding immediate recovery after mastectomy. A short length of stay and low pain scored despite a simple analgesic protocol. | 1b |
| 40 | Danielle Lovett-Carter et al, 2019 | Pectoral nerve blocks and postoperative pain outcomes after mastectomy | Breast cancer surgical patients | 7 RCT, 458 patients, met analysis | Pecs block is effective for reducing postoperative opioid consumption and pain in patients undergoing mastectomy. The Pecs block should be considered as an effective strategy to improve analgesic outcomes in patients undergoing mastectomies for breast cancer treatment. | 1a |
| 41 | Neethu M et al, 2018 | Pectoral nerve blocks to improve analgesia after breast cancer surgery | Breast cancer surgical patients | 60, RCT | Ultrasound guided combined pectoral nerve blocks are an effective modality of analgesia for patients undergoing breast surgeries during perioperative period. | 1b |
| 42 | T. Fujii et al/2019 | pectoral nerve-2 block vs. serratus plane block for chronic pain after mastectomy | Breast cancer surgical patients | 80, RCT | PECS 2 block reduced chronic pain  six months after mastectomy compared with serratus plane  block | 1b |
| 43 | Başak Altıparmak et al/2019 | Comparison of the effects of modified pectoral nerve block and erector spinae plane block on postoperative opioid consumption and pain scores of patients after radical mastectomy surgery | Breast cancer surgical patients | 38, RCT | Modified PECS block reduced postoperative tramadol consumption and pain scores more effectively than ESP block after radical mastectomy surgery. | 1c |
| 44 | Sina Grape et al/ 2020 | Analgesic efficacy of PECS and serratus plane blocks after breast surgery | Breast cancer surgical patients | 16 RCT, 1026 participants, systematic review, meta-analysis and trial sequencial analysis | PECS blocks provide postoperative analgesia after  breast surgery when compared with no regional technique and reduce rate of PONV. This might provide the most  benefit to those at high-risk of postoperative pain. | 1a |
| 45 | Sina Grape et al/ | Analgesic efficacy of PECS vs paravertebral blocks after radical mastectomy | Breast cancer surgical patients | 8 RCT 388 participants, systematic review, meta-analysis and trial sequencial analysis | There is low quality evidence that a PECS block provides marginal post-operative analgesic benefit after radical mastectomy at 2 postoperative hours only, when compared with a paravertebral block, and not beyond. | 1a |
| 46 | Boohwi Hong et al, 2021 | Comparison of PECS II and erector spinae plane block for postoperative analgesia following modified radical mastectomy | Breast cancer surgical patients | 17 RCT, 1069 Bayesian network meta‑analysis using a control group | Both PECS II and ESP  blocks were shown to be more efective than systemic analgesia regarding post-operative analgesia following modified radical mastectomy, and between the two blocks, PECS II appeared to have favorable analgesic effects compared to ESP block. | 1a |
| 47 | Narinder Pal Singh et al/ 2022 | Efficacy of regional anesthesia techniques for postoperative analgesia in patients undergoing major oncologic breast surgeries | Breast cancer surgical patients | 79 RCT, 11 interventions, 5686 participants, a systematic review and meta-analysis of randomized controlled trials | Continuous para-vertebral block and serratus anterior plane block had a high probability of reducing pain at 24 hr after major oncologic breast surgery. The certainty of evidence was moderate to very low. | 1a |
| 48 | Jessica QuinlanWoodward et al/ 2016 | Assessing the Impact of Acupuncture on Pain, Nausea, Anxiety, and Coping in Women Undergoing a Mastectomy | Breast cancer surgical patients | 30, RCT | Acupuncture adds a non-pharmacologic intervention for symptom management in women undergoing mastectomies for breast cancer. | 1c |
